# Scaffold-free bone-like 3D structure established through osteogenic differentiation from human gingiva-derived stem cells

**DOI:** 10.1016/j.bbrep.2024.101656

**Published:** 2024-02-15

**Authors:** Masaaki Toyoda, Takao Fukuda, Ryota Fujimoto, Kentaro Kawakami, Chikako Hayashi, Yuki Nakao, Yukari Watanabe, Tsukasa Aoki, Miyu Shida, Terukazu Sanui, Masahide Taguchi, Kensuke Yamamichi, Ayami Okabe, Tatsunori Okada, Kyoko Oka, Koichi Nakayama, Fusanori Nishimura, Shunichi Kajioka

**Affiliations:** a Department of Periodontology, Division of Oral Rehabilitation, Faculty of Dental Science, Kyushu University, Fukuoka, Japan; b Department of Urology, Graduate School of Medical Sciences, Kyushu University, Fukuoka, Japan; c Section of Pediatric Dentistry, Department of Oral Growth and Development, Fukuoka Dental College, Fukuoka, Japan; d Center for Regenerative Medicine Research, Faculty of Medicine, Saga University, Saga, Japan; e Department of Pharmaceutical Sciences, International University of Health and Welfare, Fukuoka, Japan; f Oral Medicine Research Center, Fukuoka Dental College, Fukuoka, Japan

**Keywords:** Human gingiva-derived mesenchymal stem cells, Bio-3D printer, Scaffold-free, Osteogenic-differentiation, Regenerative medicine

## Abstract

**Introduction & objectives:**

Stem cell therapy for regenerative medicine has been sincerely investigated, but not still popular although some clinical trials show hopeful results. This therapy is suggested to be a representative candidate such as bone defect due to the accident, iatrogenic resection oncological tumor, congenital disease, and severe periodontitis in oral region. Recently, the Bio-3D printer "Regenova®" has been introduced as an innovative three-dimensional culture system, equipped scaffold-free bio-assembling techniques without any biomaterials. Therefore, we expected a mount of bone defect could be repaired by the structure established from this Bio-3D printer using osteogenic potential stem cells.

**Material & methods:**

The gingival tissue (1x1 mm) was removed from the distal part of the lower wisdom tooth of the patients who agreed our study. Human Gingival Mesenchymal Stem Cells (hGMSCs) were isolated from this tissue and cultured, since we confirmed the characteristics such as facile isolation and accelerated proliferation, further, strong potential of osteogenic-differentiation. Spheroids were formed using hGMSC in 96-well plates designed for low cell adhesion. The size of the spheroids was measured, and fluorescent immunostaining was employed to verify the expression of stem cell and apoptosis marker, and extracellular matrix. Following four weeks of bone differentiation, μCT imaging was performed. Calcification was confirmed by alizarin red and von Kossa staining. Fluorescent immunostaining was utilized to assess the expression of markers indicative of advanced bone differentiation.

**Results:**

We have established and confirmed the spheroids (∼600 μm in diameter) constructed from human GMSCs (hGMSCs) still maintain stem cell potentials and osteogenic differentiation abilities from the results that CD73 and not CD34 were expressed as stem cell positive and negative marker, respectively. These spheroids were pilled up like cylindal shape to the “Kenzan” platform of Bio-3D printer and cultured for 7days. The cylindal structure originated from compound spheroids were tried to differentiate into bone four weeks with osteogenic induction medium. The calcification of bio-3D printed bone-like structures was confirmed by alizarin red and Von Kossa staining. In addition, μCT analysis revealed that the HU (Hounsfield Unit) of the calcified structures was almost identical to that of trabecular bone. Immunofluorescent staining detected osteocalcin expression, a late-stage bone differentiation marker.

**Conclusion:**

For the first time, we have achieved the construction of a scaffold-free, bone-like luminal structure through the assembly of spheroids comprised of this hGMSCs. This success is sure to be close to the induction of clinical application against regenerative medicine especially for bone defect disease.

## Highlights

• We developed a scaffold-free, bone-like luminal structure.
• This structure has a hardness similar to that of sponge-type bone.
• The structure has significant potential for applications in regenerative medicine.

## Graphical abstract

**Figure:**
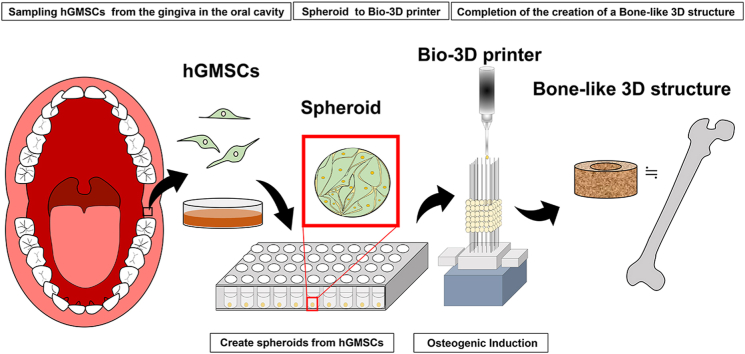

## 1 Introduction

Stem cell therapy has garnered considerable attention concomitant with advancements in regenerative medicine. However, despite rigorous research and numerous proof-of-concept studies, this discipline remains nascent. Nevertheless, clinical trials have begun to yield encouraging data, exemplified by artificial human vascular tube fabrication and nerve regeneration [1].

The efficacy of human gingiva-derived mesenchymal stem cells (hGMSCs), which are easy to isolate, is comparable with that of other stem cell types. hGMSCs proliferate rapidly independent of growth factors and maintain stable phenotypic expression, normal karyotype, and telomerase activity during protracted in vitro culture [2]. hGMSCs are advantageous for the repair of bone defects resulting from oncological resections or alveolar bone loss due to periodontitis. Such reparative processes can be achieved via minimally invasive surgical interventions within the oral cavity. Oral cancers necessitating bone resection represent an archetypal candidate for regenerative therapeutic strategies, as does severe periodontitis with alveolar bone resorption [3]. Alveolar bone resorption induces deleterious alterations, affecting nutritional status and exacerbating lifestyle-related diseases [4], thus having far-reaching implications in epidemiology and healthcare economics.

The Bio-3D printer ‘Regenova®’ is a pioneering three-dimensional (3D) culture system, allowing scaffold-free bio-assembly. This robotic automated platform manipulates the pilling of cellular spheroids affixed to a stainless steel needle array imaging BBQ style skewers closely lined up as pre-designed style [[5], [6], [7]].

Our group has focused on hGMSCs and successfully established a consistent culture strain [8]. Recently, we were granted the opportunity to use Regenova® for our studies. Investigating the regenerative capacity of hGMSCs using Bio-3D printing is vital, given their clinical implications and regeneration potential in the oral and maxillofacial regions. The objective of this study was to construct a scaffold-free 3D bone-like constructure differentiated from hGMSCs.

## 2 Material and methods

### 2.1 Ethics statement

All human gingival tissue samples used were discarded clinical specimens, obtained in accordance with an approved Institutional Review Board (IRB) protocol at the University of Pennsylvania and Kyushu University Hospital (Fukuoka, Japan). The procedures using human samples were conducted according to the Declaration of Helsinki and approved by the Kyushu University Institutional Review Board for Human Genome/Gene Research (2019-374). Written informed consents were obtained from all participants.

### 2.2 Cell culture and reagents

The gingival tissue (1x1 mm) was removed under local anesthesia from the distal part of the lower wisdom tooth of the patients who agreed to our study. hGMSCs were isolated and cultured as described previously [[9], [10], [11]]. Nucleated cells were seeded on 100-mm culture dishes with complete media containing the Alpha modification of Eagle's medium (α-MEM; Invitrogen, Waltham, MA, USA) supplemented with 10% foetal bovine serum (FBS; Hyclone Laboratories, USA), 2 mM l-glutamine (Invitrogen, Carlsbad, CA, USA), 10 mM l-ascorbic acid phosphate (Fujifilm Wako, Japan), and 100 U/mL penicillin/streptomycin (Gibco Life Technologies), followed by incubation for 48 h at 37 °C under 5% CO_2_ and 95% humidity. The cultures were washed twice with PBS to eliminate non-adherent cells. The attached cells were further cultured for another 12 days under the same conditions in the complete medium mentioned above. Cells in the 3rd–5th passages were used in subsequent experiments.

### 2.3 Quantitative RT-PCR analysis

Total RNA was isolated from the cells using ISOGENⅡ (Nippon Gene, Tokyo, Japan), and first-strand cDNA was synthesized using PrimeScript RT Master Mix (Takara Bio, Otsu, Japan). qRT-PCR was performed using the Luna Universal qPCR Master Mix (NEW ENGLAND BioLabs Inc.) on a StepOnePlus Real-Time System (Applied Biosystems, Carlsbad, CA, United States) under the following conditions: 95 °C for 1 min, 40 cycles of 95 °C for 15 s, and 60 °C for 30 s. Primer sequences used in this study are listed in Supplementary Table 1.

### 2.4 Spheroid formation

Cells were cultured in non-adhesion plates (PrimeSurface 96U Plate; Sumitomo Bakelite, Tokyo, Japan) in 2 mL α-MEM supplemented with 10% FBS, 2 mM l-glutamine, 10 mM l-ascorbic acid phosphate, and 100 U/mL penicillin/streptomycin or fibroblast growth medium (FGM™-2 BulletKit™; Lonza, Basel, Switzerland). FGM comprised fibroblast basal medium (Lonza) containing insulin, recombinant human basic fibroblast growth factor, GA-100, and FBS. The medium was changed every 3 days. GMSC spheroids were cultured in a humidified atmosphere with 5% CO_2_ at 37 °C. The diameter of each spheroid colony was measured using a bio-3D printer (Cyfuse Biomedical K.K., Tokyo, Japan) at 24, 48, and 72 h after harvest (n = 16).

The size and roundness of the spheroids were measured using Bio-3D printer software (Cyfuse Biomedical K.K., Tokyo, Japan).Roundness was calculated as follows: Circle rate (%) = 100 − (R − r)/R × 100, where ‘R’ and ‘r’ are the radius of the minimum circumscribed circle and inscribed circles, respectively.

### 2.5 Immunocytochemistry

hGMSC spheroids and scaffold-free 3D hGMSC constructure were fixed in 4% paraformaldehyde (PFA) for 30 min and washed twice with PBS before being embedded in optimal cutting temperature (OCT) compound for cryosection cutting (10-μm-thick). Cryosections of hGMSC spheroids fixed with 4% PFA were blocked for 1 h at 25 °C in Blocking One (Nacalai Tesque), and then incubated overnight at 4 °C with the following primary antibodies: vimentin (mouse IgG, 1:250; Santa Cruz Biotechnology, Dallas, TX, USA), cleaved caspase-3 (rabbit IgG, 1:250; Cell Signaling Technology, Danvers, MA, USA), CD73 (mouse IgG, 1:250; Gene Tex, Irvine, CA, USA), vimentin (rabbit IgG, 1:250; Santa Cruz Biotechnology), fibronectin (rabbit IgG, 1:200; Santa Cruz Biotechnology), and osteocalcin (rabbit IgG, 1:250; Santa Cruz Biotechnology). After washing with PBS, the cells were incubated with the appropriate secondary antibodies at room temperature for 1 h: Alexa Fluor 594 goat anti-rabbit IgG (1:250; BioLegend, San Diego, CA, USA) and Alexa Fluor 488 goat anti-mouse IgG (1:250; Cell Signaling Technology). The nuclei were stained with 4′,6-diamidino-2-phenylindole (Thermo Fisher Scientific). Coverslips were mounted using PermaFluor Mounting medium (Thermo Fisher Scientific) and images were analysed using an LSM 700 confocal microscope (Carl Zeiss) and Zen 2012 software.

### 2.6 Scaffold-free 3D GMSC construct fabrication

A scaffold-free 3D GMSC construct was fabricated using a bio-3D printer (Regenova; Cyfuse Biomedical K.K.) and needle array (hollow 9 × 9 (10 mm long) Kenzan; Cyfuse Biomedical K.K.).

We inoculated 4.0 × 10^4^ cells per well in four 96-well non-adhesion plates (PrimeSurface 96U Plate) and cultured them. After 72-h incubation, the cells formed approximately 550-μm spheroids. These spheroids were then arranged on the Kenzan array using the bio-3D printer and printed according to pre-designed architectures using the B3D designer software (Cyfuse Biomedical K.K.) [12].

### 2.7 Scaffold-free osteogenically induced construct fabrication

The prepared structures were incubated for 1 week; upon confirmation of spheroid fusion, induction was initiated in MSC osteogenic differentiation medium (Lonza) for 4 weeks. The medium was replenished every 3 days.

### 2.8 Microcomputed tomography

The fabricated spheroids and constructs were imaged in 96-well plates using microcomputed tomography (μCT) (3D Micro X-ray CT Lab GX130; Rigaku Corporation, Yamanashi, Japan) with the following parameters: tube voltage, 90 kV; tube current, 61 μA; field of view, 72 mm; and high resolution (9 μm/pixel). Images were captured at the speed of one revolution around the sample for 4 min. A bone phantom (Ratoc System Engineering, Tokyo, Japan) was used as the control. The CT values of the depicted structures were determined by setting the lower limit for obtaining 3D images in the CT Lab GX130 system (CT values of the bones ranged from 100 to 1000 Hounsfield units [HU]). The CT values were acquired with calibration settings of −1000 HU for air and 20 HU for the acrylic plate, according to the instructions of CT Lab GX130.

### 2.9 Statistical analysis

Numerical data are shown as the mean ± SD with the number of cells. Difference between means was evaluated using one-way ANOVA.

## 3 Results

### 3.1 hGMSC spheroid formation

We previously verified calcification using gingival stem cells in 2D cultures and used these cells here [8]. After confirming that hGMSCs successfully differentiated into osteogenic cells, we explored the feasibility of using gingival stem cells for spheroid fabrication. However, mechanisms underlying the osteogenic differentiation of hGMSCs remain unknown. Therefore, mRNA expression of different transcription factors was measured during each differentiation stage. When hGMSCs were cultured in ODM containing various concentrations of [Ca^2+^]_out_, *BMP2* and *Runx2* expression increased in a [Ca^2+^]_out_-dependent manner, although the Ca^2+^-sensing receptor (CaSR) agonist cinacalcet had no effect on *BMP2* expression but significantly increased *Runx2* expression after 6 h of culture in osteo-induction medium (Lonza) (Supplemental Figs. 1A and B). After 7 days of culture, *Runx2* expression decreased in a [Ca^2+^]-dependent manner and alkaline phosphatase (*ALP*) expression was significantly upregulated. After 14 days of culture, osteocalcin level increased in all [Ca^2+^]_out_-increased media and in the presence of cinacalcet.

**Fig. 1:**
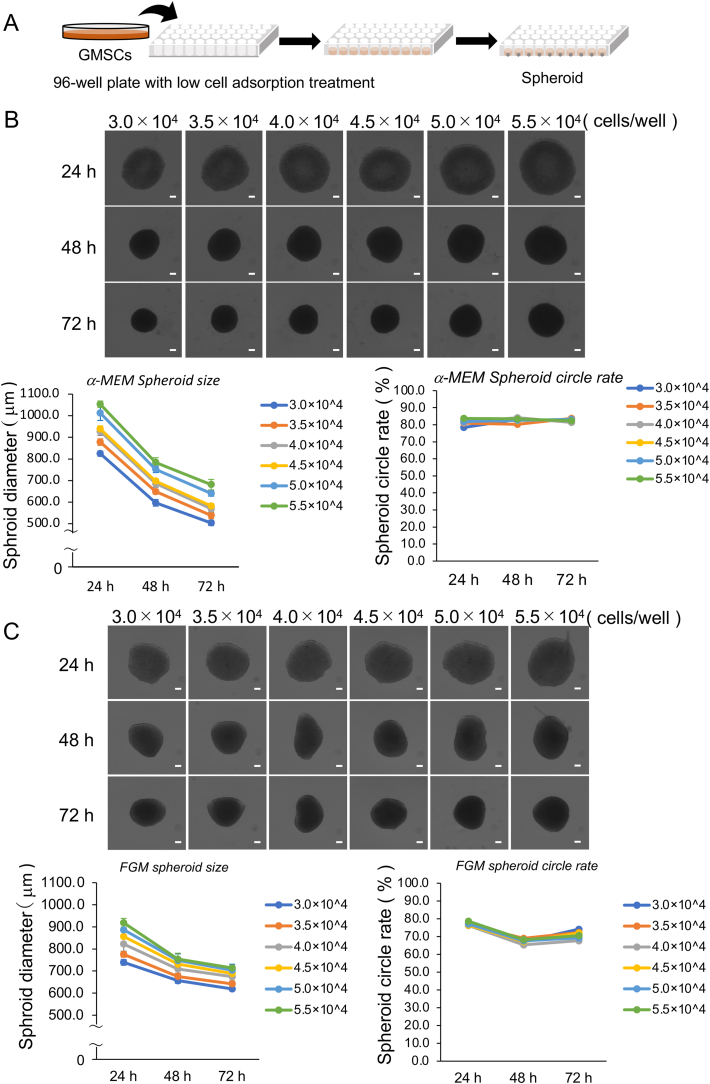
Characteristics of spheroids derived from hGMSCs. (A) Schematic representation of hGMSC spheroid formation. (B and C) Micrographs of spheroids originating from hGMSCs, with cell densities of 3.0–5.5 × 10^4^ cells/well. The cells were cultured in α-MEM and FGM. Inserted scale bar: 100 μm. Graphical displays show the spheroid diameter and circularity assessed from 24 to 72 h. Error bars represent standard error (n = 16). hGMSCs, human gingiva-derived mesenchymal stem cells; FGM, fibroblast growth medium.

Cell suspensions were prepared from monolayer cultures of gingival stem cells (Fig. 1A). Spheroids with cell densities of 3.0–5.5 × 10^4^ cells/well were generated from both FGM and α-MEM to compare their properties. Spheroids prepared in α-MEM demonstrated roundness values of 81.4%–83.7% and dimensions of 503.3–680.8 μm; those generated in the construct culture medium displayed roundness values of 69.3%–74.2% and dimensions of 619.0–713.8 μm (Fig. 1B and C). For the 9 × 9 Kenzan array, the optimal spheroid size is 500–600 μm [6]. Accordingly, spheroids with a cell density of 4.0 × 10^4^ cells/well cultured in α-MEM were selected. After 72 h, the spheroids measured 569.5 μm in size and had a roundness value of 81.4% (Supplementary Table 2).

### 3.2 Spheroid characteristics

**Fig. 2:**
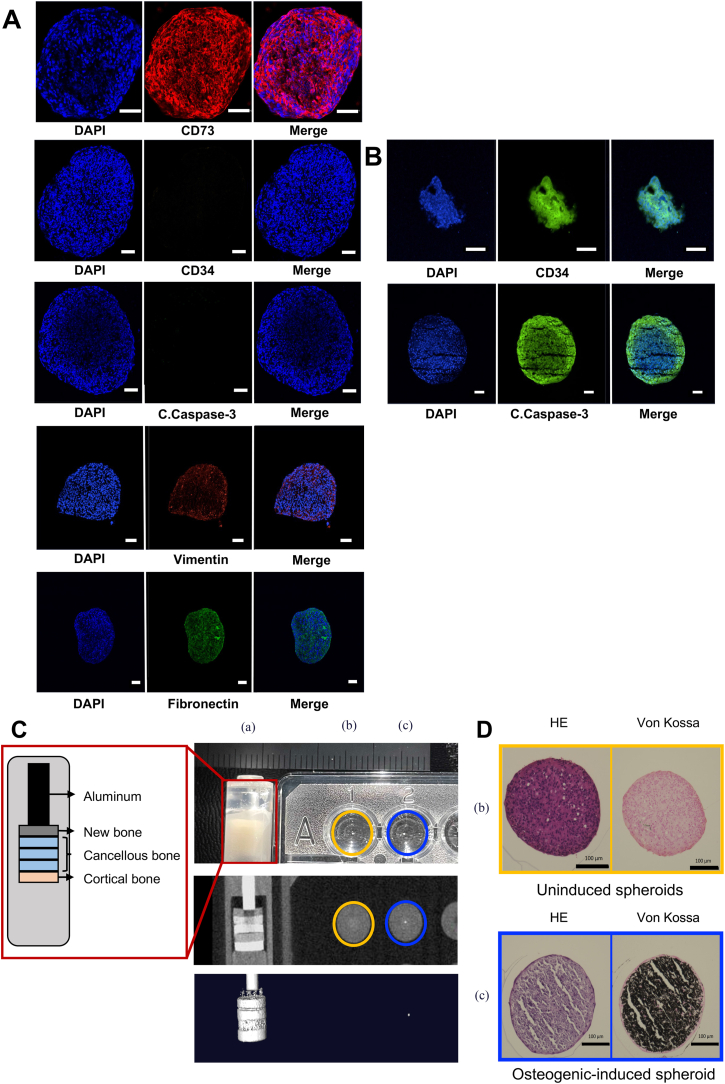
Immunofluorescence staining of hGMSCs and HUVEC spheroids. (A) Spheroids derived from hGMSCs were stained for MSC-positive (CD73), MSC-negative (CD34), apoptosis (caspase-3), epithelial–mesenchymal transition (vimentin), and cell adhesion (fibronectin). (B) HUVECs were used as positive controls and primarily stained for CD34 and caspase-3. Inserted scale bar: 50 μm. hGMSC, human gingiva-derived mesenchymal stem cell; HUVEC, human umbilical vein endothelial cell; DAPI, 4′,6-diamidino-2-phenylindole. Assessment of osteogenesis induced and non-induced spheroids with a hydroxyapatite phantom. (C) Micro-computed tomography (μCT) was employed to quantify the calcification of osteogenic-induced (blue circle: c) and non-induced spheroids (yellow circle: b). For scaling purposes, a hydroxyapatite bone bone-mineral determination phantom (a) was imaged concurrently with its schematic representation. (D) Histological sections stained with HE and von Kossa for osteogenic-induced (blue squares) and non-induced spheroids (yellow squares). Scale bar, 100 μm. DAPI, 4′,6-diamidino-2-phenylindole; HE, haematoxylin and eosin. (For interpretation of the references to colour in this figure legend, the reader is referred to the Web version of this article.)

The stem cell-positive marker CD73 and stem cell-negative marker CD34 were detected and not detected, respectively, confirming the maintenance of stem cell capacity during spheroid production. Vimentin expression in the frozen slices indicated the potential for mesenchymal differentiation into bone tissue. Fibronectin expression demonstrated the ability of the spheroids to fuse with one another (Fig. 2A and B).

In μCT, the average reaction intensity of the osteodifferentiated spheroids was 551 HU; some parts showed a maximum of 868 HU. Thus, spheroids with CT values similar to those of bone can be generated (Fig. 2C). HE staining confirmed spheroid fusion, whereas von Kossa staining revealed homogeneous calcification (Fig. 2D).

These results demonstrate that spheroids derived from hGMSCs are capable of undergoing calcification through the process of osteogenic induction.

### 3.3 Fabrication of bone-like structures

Spheroids derived from hGMSCs were cultured for 3 days and stacked on a stainless-steel needle array. Following confirmation of spheroid fusion, the spheroids were extracted and subjected to a 4-week osteogenic induction medium to generate bone-like structures.

To allow perfusion culture and culture medium circulation, the structure was designed in a hollow, cylindrical shape (Fig. 4A). After 7 days of stacking and culturing the spheroids on the needle array, fusion was confirmed, enabling the removal of the cell-derived structure from the array. The structure was transferred to the cover of the Surflo indwelling needle (14G: 2.1-mm diameter). The culture medium was replaced with a bone differentiation-induction medium to initiate bone differentiation.

**Fig. 3:**
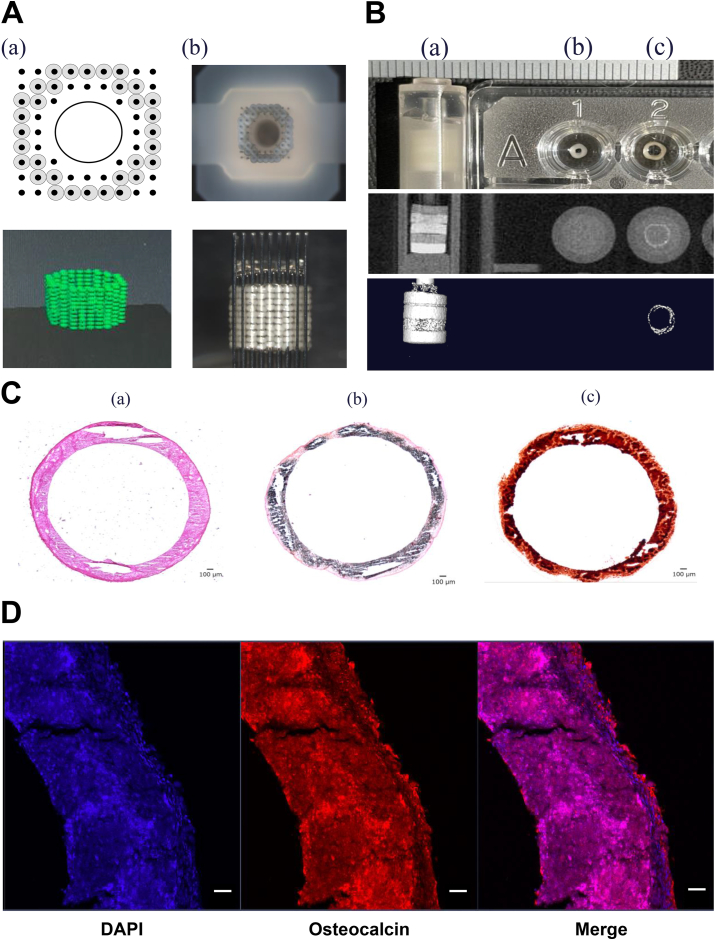
Bio-3D printing of scaffold-free osteogenic constructs derived from hGMSC spheroids. (A) Predesigned spheroids visualised from a horizontal perspective (top) and the resulting vertically oriented 3D structure after bioprinting (a). Images of the needle array (Kenzan) captured directly from above (upper), and a side view illustrating the spheroids adhered to Kenzan (lower) (b). (B) μCT scans depicting the scaffold-free osteogenic (c) and non-osteogenic constructs (b) in comparison with a hydroxyapatite bone mineral determination phantom (a) used for scaling. The middle and bottom images represent 2D and 3D perspectives, respectively. (C) Histological sections of the completed luminal 3D construct stained with haematoxylin and eosin (HE) (a), von Kossa (b), and Alizarin Red (c). (D)The presence of osteocalcin, as visualised using immunofluorescence, confirmed osteogenic differentiation. Inserted scale bar: 100 μm. hGMSC, human gingiva-derived mesenchymal stem cell. (For interpretation of the references to colour in this figure legend, the reader is referred to the Web version of this article.).

μCT imaging showed that the bone-like structures had an average CT value of 641 HU, indicating a similarity to the CT values of spongy bone (Fig. 3B).

**Fig. 4:**
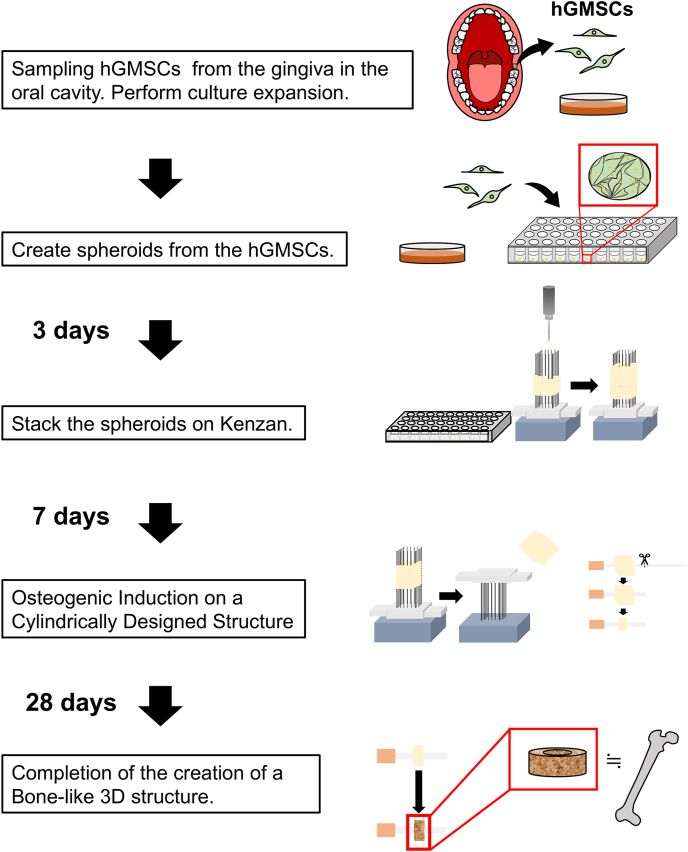
Schematic representation of the Regenova 3D bioprinting procedure for creating scaffold-free osteogenic constructs using hGMSC spheroids..

HE staining of osteodifferentiated structures demonstrated spheroid fusion; Alizarin Red and von Kossa staining revealed uniform calcification (Fig. 3C)

Subsequently, to further investigate the bone-like structures, frozen slices were prepared, and immunofluorescence staining was performed to examine the expression of Osteocalcin, a late-stage bone differentiation marker. Uniform expression of Osteocalcin was observed throughout these structures. This finding confirms that the bone-like structures have undergone substantial bone differentiation(Fig. 3D).

## 4 Discussion

We constructed a scaffold-free, bone-like luminal structure through the assembly of spheroids comprising human gingival stem cells. The hardness of the structure is similar to that of sponge-type bone, indicating its potential for clinical applications [12].

Scaffolds had been considered indispensable for cell adhesion, proliferation, and differentiation, and normal bone structure formation in regenerative medicine. However, the introduction of Regenova® [6] has revolutionised this field with its innovative 3D-culture system, which employs scaffold-free bio-assembly. This approach has been successfully applied to various organ types, including smooth muscle and cardiac, hepatic, and bone-like organs. However, this technology is limited to a few locations worldwide [[13], [14], [15]].

In tissue engineering and regenerative medicine, cells require multiple passages, which could lead to cellular fragility and reduced multipotent differentiation potential [16]. hGMSCs can be harvested from the oral mucosal epithelium, resulting in minimal trauma during collection, including painless and few bleeding. These cells exhibit excellent proliferative potential driven by their rapid turnover rates. Their stem cell capacity remains intact even after multiple passages [2]. These cells also exert an inhibitory effect on post-transplant rejection response [17].

The osteodifferentiation of spheroids comprising hGMSCs has been documented [18]. However, the underlying mechanisms remain unknown. Our immunocytochemical studies revealed that spheroids generated from hGMSCs retained their stem cell potential. They exhibited a propensity towards non-necrosis, demonstrated by the presence of the extracellular matrix marker fibronectin and epithelial–mesenchymal transition marker vimentin, as well as reduced expression of the apoptotic marker caspase-3. Based on the 3D osteogenic differentiation results, the hGMSC spheroids successfully adhered to the stainless-steel needle array of the bio-3D printer, forming a bone-like structure. Osteogenic differentiation commenced after the removal of the bio-3D printer. The osteogenic transformation of this structure was corroborated by the expression of osteocalcin, μCT imaging value of 641 HU, and HE and von Kossa staining. Therefore, the structure was sufficiently transformed into a bone matrix.

Our findings also demonstrated that α-MEM is preferable to FGM, even in the absence of growth factors, as determined by spheroid morphology, diameter, and sphericity. Human gingiva-derived stem cells cultured in α-MEM yield spheroids characterised by high aggregation and roundness. As many spheroids are required for structure fabrication using a bio-3D printer, the spheroids should be fabricated without expensive additives. After transplanting the spheroids into the bio-3D printer, the medium (specifically osteogenic differentiation medium) initially necessitates 100 mL, followed by a 50-mL infusion every 3 days for 4 weeks.

To enhance calcification, one would consider employing bone differentiation-inducing factors including BMP-2 [19,20] and TGF-β [21,22]. These factors are critical in promoting the *trans*-differentiation of non-osteogenic mesenchymal cells, including myogenic cells [23], fibroblasts [24], and adipose stem cells (ASCs) [25], into osteogenic cells; however, their high cost and potential risks of antigenicity and viral infection are problematic. An increase in the extracellular calcium ion concentration ([Ca^2+^]_out_) results in a transient increase in the intracellular calcium ion concentration ([Ca^2+^]_in_) through CaSR activation. This elevated [Ca^2+^]_in_ level stimulates BMP-2 mRNA and protein expression, suggesting that the increase in [Ca^2+^]_out_ effectively mimics the role of exogenous BMP-2 supplementation, offering a cost-effective and virus-free alternative [26]. Furthermore, the extracellular application of inorganic phosphate in conjunction with elevated calcium concentrations induces calcification in an arteriosclerosis model employing a bio-3D printer [27].

The augmentation of [Ca^2+^]_out_ triggers osteogenic differentiation of 3D structures induced by hGMSCs, providing an economically advantageous strategy. This assertion is supported by theoretical considerations and empirical evidence, as demonstrated by a 2D analysis of enhanced calcification in hGMSCs resulting from [Ca^2+^]_out_ and CaSR upregulation in hGMSCs (Supplemental Fig. 1).

We successfully engineered scaffold-free osteomimetic luminal structures that were differentiated from hGMSc (Fig. 4). However, technical and financial challenges persist in the clinical application of 3D bioprinters, namely, the time-consuming process of transplanting spheroids from 96-well plates onto a needle array and large volumes of medium required for this procedure. To address these challenges, we prepared a solution to induce osteogenic differentiation of ASCs [26], involving high-calcium culture medium instead of expensive bone-differentiation-inducing factors. Regarding material and the adherence of stem cells, it has been successfully attached to titanium as a non-biomaterial in short-term studies. However, long-term validation has not yet been conducted, representing an area for future research. As biomaterials, those derived from bovine and pig sources are commonly utilized. However, the risk of antigenicities and infectious disease transmission associated with these materials is an unavoidable concern [28,29]. Our success in this endeavour led us to explore the application of this technology to human gingiva-derived stem cells, offering promising results for further advancement.

## Funding

This work was supported by a Grants-in-Aid for Scientific Research from 10.13039/501100001691JSPS [grant numbers JP20H03865, JP21K21017, and JP22K21041 (T F, Y.N, and Y.W)] and JST SPRING [grant number JPMJSP2136].

## Data statement

Data supporting the findings of this study are available in the article and its supplementary materials.

## CRediT authorship contribution statement

**Masaaki Toyoda:** Conceptualization, Data curation, Formal analysis, Funding acquisition, Investigation, Methodology, Project administration, Resources, Software, Validation, Visualization, Writing – original draft, Writing – review & editing. **Takao Fukuda:** Supervision. **Ryota Fujimoto:** Project administration, Resources, Methodology. **Kentaro Kawakami:** Data curation, Formal analysis, Investigation. **Chikako Hayashi:** Formal analysis, Investigation, Methodology. **Yuki Nakao:** Data curation, Formal analysis, Funding acquisition. **Yukari Watanabe:** Data curation, Formal analysis. **Tsukasa Aoki:** Data curation, Formal analysis. **Miyu Shida:** Investigation. **Terukazu Sanui:** Project administration. **Masahide Taguchi:** Data curation, Investigation. **Kensuke Yamamichi:** Project administration. **Ayami Okabe:** Data curation, Investigation. **Tatsunori Okada:** Data curation, Investigation. **Kyoko Oka:** Project administration. **Koichi Nakayama:** Project administration. **Fusanori Nishimura:** Project administration. **Shunichi Kajioka:** Conceptualization, Data curation, Formal analysis, Funding acquisition, Investigation, Methodology, Project administration, Resources, Software, Supervision, Validation, Visualization, Writing – original draft, Writing – review & editing.

## Declaration of competing interest

The all authors declare that they have no known competing financial interests or personal relationships that could have appeared to influence the work reported in this paper.

## Data Availability

No data was used for the research described in the article.
