# Supplementary material for: Scaffold-free bone-like 3D structure established through osteogenic differentiation from human gingiva-derived stem cells
Source: Biochem Biophys Rep. 2024 Feb 15;38:101656. doi: 10.1016/j.bbrep.2024.101656 (PMC10878834; doi:10.1016/j.bbrep.2024.101656)
Supplement: Multimedia component 1 [file mmc1.docx]

Supplementary Material

# Supplementary Figure 1.

A) The BMP2 mRNA expression of hGMSCs after 6-hour culture in the medium involving various concentrations of Ca2+ (3, 5, and 10 mM) or the CaSR agonist cinacalcet.

(B) The Runx2 mRNA expression of hGMSCs after 6-hour culture in the medium involving various concentrations of Ca2+ (3, 5, and 10 mM) or the CaSR agonist cinacalcet.

(C) The Runx2 mRNA expression of hGMSCs after 7-day culture in the medium involving various concentrations of Ca2+ (3, 5, and 10 mM) or the CaSR agonist cinacalcet.

(D) The ALP mRNA expression of hGMSCs after 7-day culture in the medium involving various concentrations of Ca2+ (3, 5, and 10 mM) or the CaSR agonist cinacalcet.

(E) The osteocalcin mRNA expression of hGMSCs after 14-day culture in the medium involving various concentrations of Ca2+ (3, 5, and 10 mM) or the CaSR agonist cinacalcet.

**Supplementary Table 1.**

Primer sequence used for quantitative RT-PCR

| **Gene** | **Forward primer** | **Reverse primer** |
| --- | --- | --- |
| ***human Runx2*** | 5'-**GCGTCAACACCATCATTCTG**-3' | 5'-**CAGACCAGCAGCACTCCATC**-3' |
| ***human ALP*** | 5'-**GACAAGAAGCCCTTCACTGC**-3' | 5'-**AGACTGCGCCTGGTAGTTG**-3' |
| ***human OCN*** | **5'-GGCGCTACCTGTATCAATGG-3'** | **5'-TCAGCCAACTCGTCACAGTC -3'** |
| ***human BMP-2*** | 5'-CCCTACATGCTAGACCTGTATCG-3' | 5'-**TCCTCCGTGGGGATAGAAC** 3' |
| ***human GAPDH*** | 5'-**ATCAAGAAGGTGGTGAAGCAGG**-3' | 5'-**GTCATACCAGGAAATGAGC**-3' |

# Supplementary Table 2.

|  | α-MEM Spheroid size | |  |  |  |  |
| --- | --- | --- | --- | --- | --- | --- |
|  | 3.0×10^4 cells/well | 3.5×10^4 cells/well | 4.0×10^4 cells/well | 4.5×10^4 cells/well | 5.0×10^4 cells/well | 5.5×10^4 cells/well |
| 24 h | 824.5 (SD±12.0) | 877.9 (SD±15.3) | 927.8 (SD±18.6) | 938.4 (SD±14.8) | 1012.0 (SD±33.8) | 1053.1 (SD±15.0) |
| 48 h | 596.9 (SD±15.6) | 648.5 (SD±11.7) | 686.2 (SD±17.1) | 696.9 (SD±11.7) | 751.9 (SD±15.2) | 783.6 (SD±22.0) |
| 72 h | 503.3 (SD±12.0) | 538.3 (SD±15.1) | 569.5 (SD±15.5) | 582.5 (SD±11.5) | 640.2 (SD±12.7) | 680.8 (SD±24.2) |
|  |  |  |  |  | n＝16 | (mm) |
|  | α-MEM Spheroid circle rate | |  |  |  |  |
|  | 3.0×10^4 cells/well | 3.5×10^4 cells/well | 4.0×10^4 cells/well | 4.5×10^4 cells/well | 5.0×10^4 cells/well | 5.5×10^4 cells/well |
| 24 h | 78.4 (SD±4.9) | 81.0 (SD±3.9) | 81.4 (SD±4.1) | 82.6 (SD±4.9) | 81.9 (SD±7.0) | 83.7 (SD±5.1) |
| 48 h | 83.2 (SD±4.1) | 80.3 (SD±4.8) | 84.1 (SD±2.8) | 82.4 (SD±3.5) | 82.6 (SD±5.0) | 83.4 (SD±4.0) |
| 72 h | 82.2 (SD±7.8) | 83.7 (SD±5.1) | 81.4 (SD±5.7) | 83.3 (SD±4.3) | 83.2 (SD±4.3) | 82.2 (SD±3.6) |
|  |  |  |  |  | n＝16 | (%) |
|  | FGM Spheroid size | |  |  |  |  |
|  | 3.0×10^4 cells/well | 3.5×10^4 cells/well | 4.0×10^4 cells/well | 4.5×10^4 cells/well | 5.0×10^4 cells/well | 5.5×10^4 cells/well |
| 24 h | 739.3 (SD±12.9) | 775.6 (SD±13.4) | 822.2 (SD±32.4) | 856.4 (SD±27.9) | 886.9 (SD±22.5) | 918.6 (SD±19.6) |
| 48 h | 656.7 (SD±12.7) | 675.4 (SD±15.1) | 709.0 (SD±21.8) | 731.1 (SD±22.4) | 748.4 (SD±29.2) | 753.7 (SD±27.0) |
| 72 h | 619.0 (SD±9.4) | 641.8 (SD±14.0) | 674.7 (SD±21.2) | 688.8 (SD±21.8) | 703.6 (SD±27.3) | 713.8 (SD±11.8) |
|  |  |  |  |  | n＝16 | (mm) |
|  | FGM Spheroid circle rate | |  |  |  |  |
|  | 3.0×10^4 cells/well | 3.5×10^4 cells/well | 4.0×10^4 cells/well | 4.5×10^4 cells/well | 5.0×10^4 cells/well | 5.5×10^4 cells/well |
| 24 h | 77.2 (SD±3.9) | 76.8 (SD±4.5) | 76.3 (SD±5.0) | 76.5 (SD±4.8) | 77.2 (SD±5.0) | 78.7 (SD±4.5) |
| 48 h | 67.1 (SD±7.4) | 69.1 (SD±12.9) | 65.4 (SD±9.5) | 67.2 (SD±10.4) | 67.6 (SD±9.6) | 68.3 (SD±11.3) |
| 72 h | 74.2 (SD±8.8) | 72.4 (SD±7.5) | 67.7 (SD±11.6) | 72.0 (SD±11.1) | 69.3 (SD±12.6) | 70.6 (SD±9.4) |
|  |  |  |  |  | n＝16 | ( % ) |

Optimal conditions (number of seeded cells, medium) for spheroid culture in α-MEM and FGM, compared by size and circle rate.
